# Utility of transcranial Doppler and reversed jugular venous saturations for neuromonitoring in children with acute liver failure

**DOI:** 10.1007/s00431-025-06697-2

**Published:** 2025-12-24

**Authors:** Raman Singla, Emma C. Alexander, Pervez Khan, Ben Freedman, Rehab Elseidy, Subramanyam Sheshadri, Bogdana Zoica, Akash Deep

**Affiliations:** 1 Division of Paediatric Intensive Care, King’s College Hospital NHS Foundation Trust, Denmark Hill, London, SE5 9RS UK; 2 Liver Intensive Therapy Unit, Institute of Liver Studies, King’s College Hospital, London, UK; 3 Vascular Laboratory, King’s College Hospital, London, UK; 4 Department of Women and Children’s Health, School of Life Course Sciences, King’s College London, London, UK

**Keywords:** Paediatric acute liver failure (PALF), Transcranial doppler (TCD), Reverse jugular venous saturation (SjvO_2_), Neuromonitoring

## Abstract

Paediatric Acute Liver Failure (PALF) is associated with high morbidity and mortality, with cerebral oedema a key contributor to mortality. The aim of our study was to examine the association between the findings of two neuromonitoring modalities (transcranial Doppler (TCD) and reverse venous jugular oxygen saturation (SjvO_2_) and mortality, as well as ammonia levels, in children with acute liver failure who received neuroprotection within 7 days of paediatric intensive care unit (PICU) admission. Retrospective observational cohort study. Sixteen-bed PICU in a UK university-affiliated tertiary care hospital. Patients admitted to the PICU with PALF were reviewed between January 2013 and October 2023. Seventy-eight children, who were intubated and ventilated, had neuromonitoring either with TCD/SjvO_2_ or both during the first week of hospital admission (before liver transplantation), and were included in the study. Interventions: None. The median (IQR) age of the study population was 64 (25–144) months. Among the 78 children, 24 (30.8%) survived without liver transplant, 39 children (50.0%) survived post-liver transplant, 5 (6.4%) died post-transplant, and 10 (12.8%) died without transplant. Patients who died had a lower meanvelocity in the right middle cerebral artery (RMCA Vm) (Median: 34 cm/s vs 55 cm/s, *p*-value: 0.01) and higher pulsatility index (PI) than those who survived (Median: 2.1 vs 1.6, *p*: 0.001). SjvO_2_ was also lower in non-survivors than in survivors (Mean: 39.0% vs 56.1%, *p*-value: 0.002). The PI had a weak positive correlation with ammonia (Rho: 0.25, *p*: 0.005). RMCA Vm and SjvO_2_ had a negative correlation with ammonia (Rho: − 0.21, *p*-value: 0.022; Rho − 0.22; *p*-value: 0.017 respectively). *Conclusion*: Patients with acute liver failure who died had a high RMCA PI, lower RMCA Vm, and lower SjvO_2_. TCD and SjvO_2_ may serve as useful adjuncts to the overall neurological assessment in critically ill children with PALF.

**Table:** 

**What is Known:** • *Paediatric Acute Liver Failure (PALF) is associated with high morbidity and mortality, with cerebral oedema a key contributor to mortality.* • *There is little published evidence regarding the use of bedside neuromonitoring techniques like transcranial dopplers and reverse jugular saturations in the paediatric population with acute liver failure.*	
**What is New:** • *In our single-centre experience (2013–2023), neuromonitoring in PALF patients correlates with mortality. Bedside neuromonitoring with TCD and SjvO2 showed a weak correlation with ammonia and between each other.* • *Future work should focus on the impact of neuromonitoring on delivered neuroprotective strategies and patient outcomes.*

**Supplementary Information:**

The online version contains supplementary material available at 10.1007/s00431-025-06697-2.

## Introduction

Paediatric acute liver failure (PALF) is a rare condition associated with high morbidity and mortality. It is defined in children by the following: (a) no known evidence of chronic liver disease, (b) biochemical evidence of acute liver injury, and (c) coagulopathy. Coagulopathy is defined as a prothrombin time (PT) ≥ 15 s or INR ≥ 1.5 not corrected by Vitamin K in the presence of clinical hepatic encephalopathy, or PT ≥ 20 s or INR ≥ 2.0 regardless of the presence or absence of clinical hepatic encephalopathy [1].

Neurological complications, in particular, cerebral oedema leading to raised intracranial pressure (ICP), occur in about 30% of cases and are leading drivers of mortality. The most important contributing factor to cerebral oedema is hyperammonemia. In PALF, impaired ureagenesis leads to ammonia accumulation. Ammonia is then metabolised into glutamine, an osmotically active molecule that contributes to cytotoxic cerebral oedema [2]. Consequently, neuromonitoring and neuroprotective measures are key to the intensive care management of PALF [3]. Grade of hepatic encephalopathy (HE) is associated with the development of cerebral oedema. However, judging the grade of HE can be challenging, especially in young, non-verbal children. Therefore, tools to detect features of raised ICP early are potentially of great value in the paediatric population [4].

Invasive ICP monitoring remains the gold standard for neuro-monitoring in critically ill patients. However, its use is associated with a potential risk of intracranial bleeding, particularly in severely coagulopathic patients. While data on the incidence of significant haemorrhagic complications in paediatric patients remain limited, concerns regarding procedural risks often favour non-invasive monitoring strategies. Emerging non-invasive modalities provide real-time, bedside assessment of cerebral haemodynamics, potentially allowing for earlier detection of intracranial hypertension and timely intervention while mitigating the risks associated with invasive ICP monitoring.

Examples of these techniques include transcranial Doppler (TCD), which has been used in adult patients with acute liver failure, to assess cerebral perfusion. Previous studies have successfully correlated TCD parameters with ICP levels [5, 6]. Similarly, studies have shown that the pulsatility index (PI) is higher in encephalopathic patients than in non-encephalopathic patients with cirrhosis [7, 8]. In children, a single-centre study in ten children treated for PALF in France between 2015 and 2019 reported the use of TCD in PALF [9].

Another example of bedside neuromonitoring is reverse jugular venous oxygen saturations (SjvO_2_), in which the right internal jugular vein is cannulated in the reverse direction towards the base of the skull, and jugular oximetry is monitored. This technique has been rarely described in children, but it has shown some association with poor neurological outcomes in cases of traumatic brain injury [10]. A 2019 systematic review by our research group identified a preponderance of adult studies, with very limited paediatric data about neuromonitoring techniques in PALF [4].

Therefore, this single-centre study aims to report the association of bedside neuromonitoring techniques (TCD and reverse jugular venous saturations) with outcomes in a cohort of patients with PALF. We also investigate the association of ammonia levels with TCD parameters, considering that high ammonia levels at illness presentation, as well as persistent hyperammonaemia, have been well-established as associated with cerebral oedema [2, 11].

## Methods

### Study participants

This study is a single-centred retrospective review of routinely collected data from January 2013 to October 2023. The project was registered as a service improvement project by the King’s College Hospital Research and Innovation team (CH-060). No institutional review board (IRB) review was necessary (and thus no number was assigned) because it did not fall under the board’s guidelines as human subjects research.

The patients were admitted to the Paediatric Intensive Care Unit (PICU) at King’s College Hospital, which serves as a supra-regional centre for liver disease in the United Kingdom. Children who met the PALFSG (Paediatric Acute Liver Failure Study Group) criteria, and were intubated, ventilated, and managed with neuroprotection and had at least one observation of transcranial Doppler (TCD) or reverse jugular venous saturation (SjvO_2_) measured within the first 7 days of admission (before liver transplant) in the unit, were included. At our institute, we follow a dedicated neuroprotection protocol that includes maintaining therapeutic normothermia, keeping CO₂ within the target range, and supporting mean arterial pressure around the 50th centile to ensure adequate cerebral perfusion (Supplementary Table 1).

Clinical data were extracted from the local electronic patient records system (electronic patient record by Sunrise EPR 18.2, Metavision by iMD Soft, and EPIC by Apollo). Baseline data, including age, sex, biochemical and clinical characteristics, PIM (Patient Index of Mortality) 3 score, and aetiology, were recorded. For each patient, the decision to perform neuromonitoring techniques was determined by the attending PICU physician.

### Neuromonitoring

In our unit, all children with PALF who require intubation and ventilation typically undergo neuromonitoring (TCD and/or SjvO_2_) according to the unit protocol. At our centre, TCDs were performed either by vascular physicians or trained paediatric intensivists at the bedside at an interval of 24 h (or earlier if clinically indicated) using the 5 MHz probe of the Phillips ultrasound machine through a transtemporal window. After identifying the middle cerebral artery, velocities and pulsatility index were measured using pulse wave (PW) Doppler on both sides. TCD data was retrieved using PACS (Picture Archiving and Communication System). As velocities and PI were comparable on both sides, the right side was used to standardise the results. No child had major focal pathology as confirmed clinically by equal pupillary size and, in case of clinical suspicion, confirmed by CT scan. In transcranial dopplers, mean velocity indicates the average velocity of blood flow in the brain, which is calculated using the peak systolic velocity (PSV) and end-diastolic velocity (EDV) based on the following formula: 1/3 * PSV + EDV. The pulsatility index is a measure of blood velocity in a vessel and is indicative of peripheral resistance which is calculated by dividing the difference between peak systolic and end diastolic velocities by the mean velocity [12]. In case of multiple values over 7 days, the maximum PI was recorded, and the corresponding mean velocity was recorded for each patient.

As per standard institutional practice, the reverse jugular venous catheter was inserted under aseptic conditions using ultrasound guidance in mechanically ventilated children older than 1 year. The Seldinger technique was used to introduce the sheath. The patency of the catheter was maintained by continuous infusion of normal saline. SjvO_2_ was measured intermittently every 4 h by the bedside PICU-trained nurse using a blood gas machine. When multiple observations were available within 24 h, only those recorded within 4 h of the transcranial Doppler and 30 min of ammonia measurement were considered. Serum ammonia levels were typically measured every 8 h. The value closest in time to the measurement was selected for each patient.

Abnormalities detected by these neuromonitoring methods (elevated RMCA PI, reduced mean velocity, or low SjvO_2_) were managed according to the local institutional guideline for raised intracranial pressure (ICP) in children with acute liver failure. These included measures such as head elevation, optimization of ventilation, blood pressure and sedation, hyperosmolar therapy and controlled hyperventilation when indicated. The decision to initiate these interventions was made by the attending PICU consultant based on the clinical picture and neuromonitoring trends.

### Outcomes

We aimed to assess the relationship between Transcranial Dopplers (TCD) variables (maximum PI and mean velocity in the right middle cerebral artery [Rmca Vm]) and minimum SjvO_2_ within 7 days of PICU admission (before liver transplant) with clinical outcomes (all-cause mortality and transplant-free survival [TFS]). In addition, we examined the correlation between TCD variables and SjvO_2_ values with corresponding ammonia values, and the correlation between TCD variables and SjvO_2_.

### Statistical analysis

Statistical analyses were performed by using Stata 14.2 (StataCorp, College Station, TX, USA). Continuous data with normal distribution were presented as mean ± standard deviation (SD), while skewed data were presented as median (inter-quartile range [IQR]). Comparisons used the Student’s *t*-test or Mann–Whitney *U* test as appropriate. Categorical data are represented as a number (%). The correlation between quantitative variables was calculated using Pearson or Spearman correlation as appropriate. Statistical significance was defined as a *p*-value < 0.05.

## Results

**Fig. 1:**
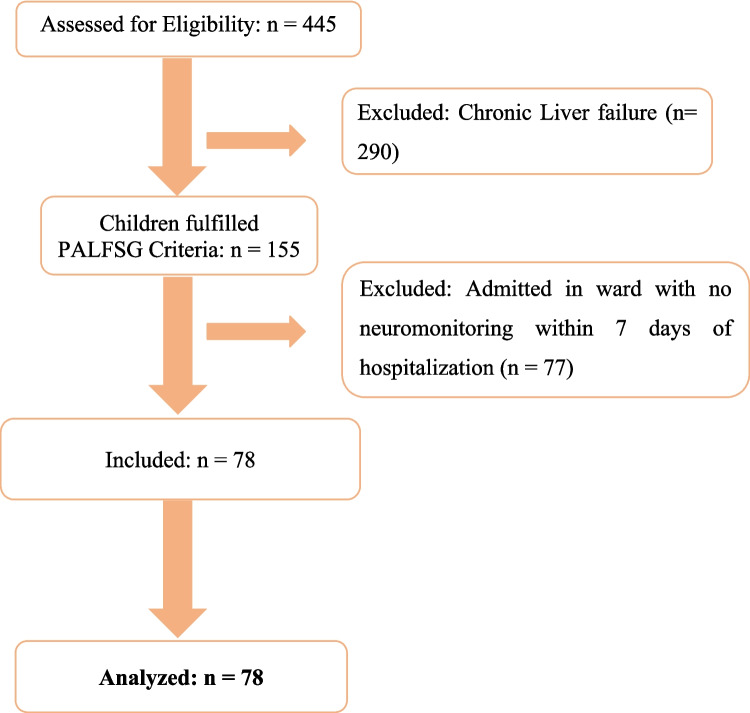
Study inclusion flow diagram

One hundred and fifty-five children met the PALF criteria between January 2013 to October 2023. Among them, 78 had neuromonitoring with either TCD or SjvO_2_ or both and were included in the study. The study flowchart is displayed in Fig. 1.

Out of 78 enrolled children, 24 (30.8%) survived without liver transplant (survived with native liver) whereas 39 children (50.0%) survived post-liver transplant. Overall, 10 (12.8%) children died without liver transplantation, and 5 (6.4%) died post-liver transplant. The median age (interquartile range [IQR]) of the enrolled population was 64 (25–144) months. Overall, 51.3% (*n* = 40) of the cohort were male. Other baseline variables are described in Table 1.

**Table 1:** Baseline demographic characteristics of included Paediatric Acute Liver Failure (PALF) patients

Characteristics	*n* = 78
Age, median (IQR), months	64 (25–144)
Gender category Male, *n* (%)	40 (51.3)
Grade of Encephalopathy at presentation, *n* (%)	
No Encephalopathy	0 (0)
Grade 1	10 (12.8)
Grade 2	17 (21.8)
Grade 3	16 (20.5)
Grade 4	35 (44.9)
Parameters at ICU Admission, median (IQR)	
PIM3 Score	0.06 (0.02–0.15)
INR (in seconds)	3.4 (2.8–4.4)
Fibrinogen (g/L)	1.2 (0.9–1.6)
Total Bilirubin (μmol/L)	175 (66–330)
Albumin (g/dL)	29 (24–32)
Ammonia (μmol/L)	107 (71–139)
Sodium (mmol/L)	142 (139–144)
Organ Support received within 7 days, *n* (%)	
Mechanical Ventilation	78 (100)
Inotropes/vasopressors	68 (87.2)
CVVH	61 (78.2)
Etiology, *n* (%)	
Viral Hepatitis	16 (20.5)
Drug overdose (including acetaminophen)	11 (14.1)
Indeterminate	27 (34.6)
Miscellaneous	24 (30.8)
Outcome, n (%)	
Survivors with Native Liver	24 (30.8)
Survivors with Transplanted Liver	39 (50.0)
Non-Survivors without transplantation	10 (12.8)
Non-Survivors after transplantation	5 (6.4)

### Relation between TCD parameters, SjvO_2_ and all-cause mortality

Neuromonitoring parameters differed between survivors and non-survivors. The median of the highest RMCA PI within 7 days of admission was 2.2 (*n* = 13; IQR 1.8–2.7) in non-survivors, whereas the same was 1.6 (*n* = 57; IQR 1.2–1.9); *p*-value: 0.001 in survivors. Similarly, patients who died had lower Vm with a median (*n*; IQR) of 34 cm/s (*n* = 13; 22 cm/s–62 cm/s) compared to those who survived with a median (*n*; IQR) of 55 cm/s (55; 45 cm/s–80 cm/s), *p*-value: 0.01. After adjusting for age, both PI and Vm maintained a significant association with mortality. While comparing the SjvO_2_ values, non-survivors had a mean saturation of 39.0% (*n* = 7; 95% CI: 31.4%–46.7%), which was significantly lower than in survivors who had a mean value of 56.1% (*n* = 36; 95% CI: 51.5%–60.6%). The mean difference was 17.0% (95% CI: 6.4%–27.7%, *p*-value: 0.002). To exclude the possible confounding factor of low haemoglobin, we analyzed the haemoglobin between the non-survivors and survivors, and no difference was demonstrated (94 g/dL, 95% CI: 81–106; 93 g/dL, 95% CI: 87–101).

### Relation between TCD parameters and SjvO_2_ and transplant-free survival

In the cohort of surviving patients, the median of peak RMCA PI in patients requiring transplantation was 1.6 (*n* = 35; IQR: 1.2–1.9), whereas non-transplanted survivors had a median of 1.4 (*n* = 22; IQR 1.2–1.8), with no significant difference (*p*-value: 0.31). Similarly, median (*n*, IQR) RMCA Vm were 50 cm/s (22; 45–71) in the transplant group and 60 cm/s (33; 50–82) in the transplant-free survivors (*p*-value: 0.19). Mean SjvO_2_ in the transplant group was 54.6% (*n* = 24, 95% CI: 41.6–67.6) and in transplant-free survivors, was 59.0% (*n* = 12, 95% CI: 44.7–72.2), *p*-value: 0.37.

### Correlation between TCD parameters and SjvO_2_ with ammonia

**Fig. 2:**
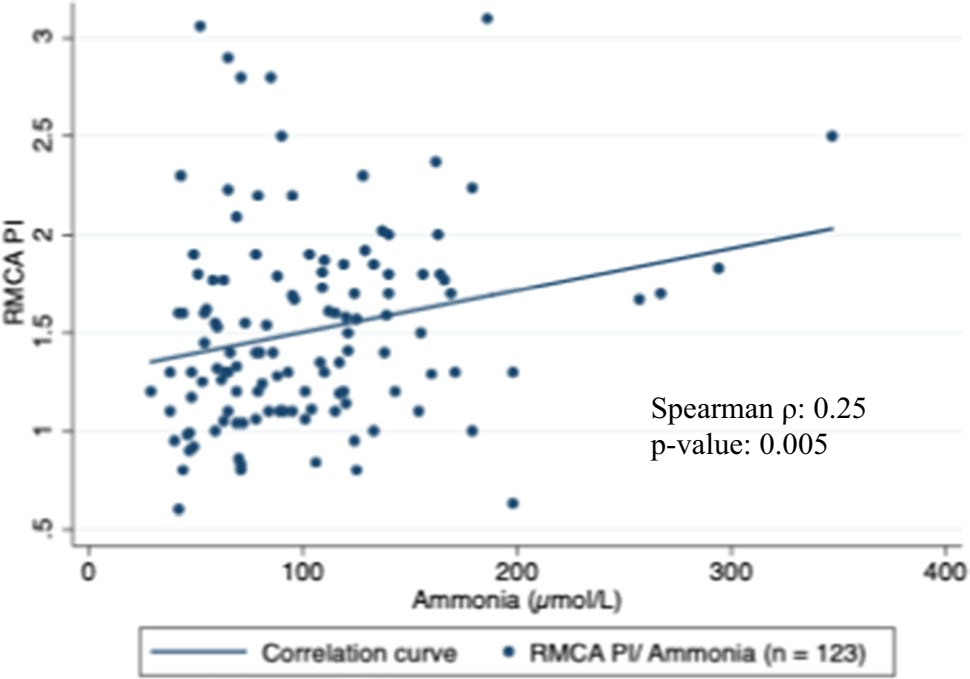
Correlation between RMCA PI and Ammonia

**Fig. 3:**
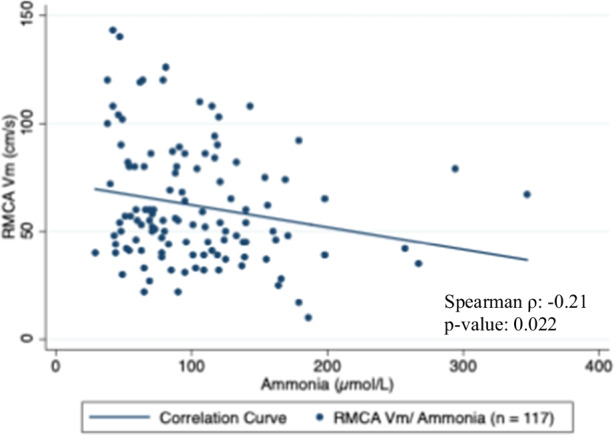
Correlation between RMCA Vm and Ammonia

**Fig. 4:**
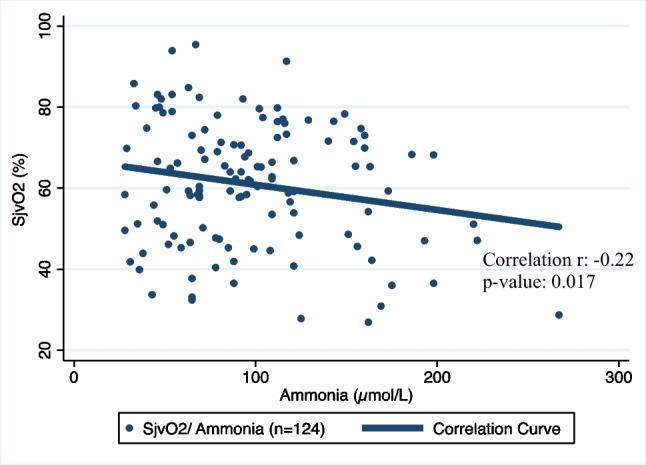
Correlation between SjvO_2_ and Ammonia

Our study revealed a weak positive correlation between the RMCA PI and ammonia with a Spearman coefficient of 0.25 (Total observations: 123; *p*-value: 0.005) (Fig. 2). RMCA Vm had a weak negative correlation with ammonia with a Spearman coefficient of −0.21 (Total observations: 117; *p*-value: 0.022) (Fig. 3). Furthermore, we found a statistically significant weak negative correlation between SjvO_2_ and simultaneous ammonia levels (Correlation coefficient: − 0.22, Total observations: 124; *p*-value: 0.017) (Fig. 4).

### Association between TCD parameters and SjvO_2_

The findings suggest that the RMCA PI negatively correlates with SjvO_2_ with a correlation coefficient of − 0.31 (Total observations: 77; *p*-value: 0.006) (Supplementary Fig. 1). However, the RMCA Vm was positively correlated with Reverse venous Jugular saturation (Pearson coefficient: 0.40; total observations 77; *p*-value: 0.0004) (Supplementary Fig. 2).

## Discussion

Cerebral oedema with raised intracranial pressure is a contributor to morbidity and mortality in PALF, but is challenging to assess in children. Adult studies suggest that improved neuromonitoring and neuroprotective measures have led to a significant decline in the incidence of cerebral oedema and intracranial hypertension in ALF [3, 13]. However, it remains a concern that invasive ICP monitoring carries a potential risk of intracranial bleeding [14]. Specific paediatric data regarding rates of significant haemorrhage is lacking — one study reported one in 14 children experienced an intracranial haemorrhage after ICP bolt insertion [15]. However, it is plausible that bedside monitoring, when used appropriately, will have fewer potential risks and have similar potential to provide real-time, bedside assessment of cerebral haemodynamics.

When intracranial pressure (ICP) rises, it exerts a compressive effect on the surrounding major vessels, such as the middle cerebral artery. This compression particularly reduces systolic blood flow, leading to an increase in the PI [16, 17]. In line with the Monro–Kellie principle, elevated ICP also reduces cerebral blood flow. When cerebral blood flow drops below the brain’s metabolic requirements, there is a compensatory increase in oxygen extraction, which results in reduced jugular venous oxygen saturation (SjvO_2_) [18].

In this study, we found a correlation between bedside neuromonitoring modalities with clinical outcomes (mortality) and ammonia levels. Compared with ammonia levels, TCD and SjvO_2_ provide real-time information on cerebral haemodynamics and oxygenation. Although ammonia is an important driver of cerebral oedema, other mechanisms such as changes in mitochondrial membrane permeability, oxidative stress also contribute [20]. As a result, ammonia levels alone may not fully reflect the extent of cerebral compromise, whereas TCD and SjvO_2_ can offer a more immediate picture of the physiological status. Hence, together these modalities can inform timely, targeted neuroprotective strategies in patients at risk of intracranial hypertension.

Our findings indicate that the non-survivors had a significantly higher RMCA PI and a lower RMCA Vm than survivors. These results align with findings in adult populations, where increased PI and decreased flow state have been observed in patients who did not survive, compared to those who either survived until transplantation or underwent transplantation [5, 7, 19]. For instance, in an adult cohort of 21 patients with acute liver failure, published in 2015, also had lower Vm and higher PI via TCD [5]. Cousin et al. also observed more loss of End Diastolic Velocity (EDV) in non-survivors (*n* = 4) in comparison to survivors (*n* = 6) in a paediatric cohort [9].

Additionally, our study suggested that non-survivors had lower SjvO_2_ than survivors. Physiologically, lower reverse venous jugular saturation is suggestive of a reduced supply to oxygen to the brain (e.g. cerebral vasoconstriction/vasospasm) or increased metabolic demand (e.g. seizures), whereas higher saturation indicates the increased supply of oxygen (e.g. cerebral vasodilation, hypertension) or reduced oxygen requirement (e.g. Coma, brain death) [21, 22]. The correlation between the SjvO_2_ and corresponding ammonia values strengthens our hypothesis. Finally, we identified a correlation between TCD and SjvO_2_. This is plausible given that both are measurements of cerebral haemodynamic compromise.

Studies in the past have mentioned the complications while inserting the SjvO_2_ catheter, such as accidental carotid artery puncture, thrombosis or haematoma formation. These complications are similar to those associated with the insertion of an internal jugular central venous catheter [23–25]. We did not separately record the complications associated with SjvO_2_ catheter insertion in our cohort, though there were no major complications (air leak, major haemorrhage requiring blood transfusion).

Finally, in our study, we did not find a difference in RMCA Vm, PI or SjvO_2_ among transplant-free survivors compared to transplant recipients. This differed from prior studies, which demonstrated a relationship between abnormal TCD values and the need for liver transplantation [5, 19]. Listing for transplant is a complex decision and involves many factors in addition to the degree of critical illness of the patient, including aetiology and psychosocial considerations, which may differ from adult patients [26].

Overall, our findings suggest that bedside neuromonitoring could be integrated into the clinical assessment of children with PALF, with acknowledgement of the overall limited evidence and its limitations. As a non-invasive, portable tool, TCD can be easily incorporated into daily practice to assess cerebral blood flow dynamics, guide neurocritical care management, and support decision-making in conditions like ALF. Training in transcranial Doppler is feasible for intensivists through focused workshops, online courses, and hands-on bedside practice. With structured training and regular use, intensivists may be able to enhance neuro-monitoring capabilities and improve patient outcomes. Similarly, inserting a reverse jugular venous catheter under ultrasound guidance is a relatively easy skill in comparison to insertion of an ICP bolt. Monitoring of SjvO_2_ may be more feasible than TCD monitoring in centres where TCD is not readily available, given that all ICUs should have the capability to insert central venous lines. Other neuromonitoring techniques worthy of further research include optic nerve sheath diameter (ONS), amplitude-integrated electroencephalography (aEEG), near-infrared spectroscopy (NIRS) and tympanic membrane displacement (TMD) [4].

In terms of strengths, to our knowledge, our study represents the largest PALF cohort describing the utility of bedside neuromonitoring. However, there are several limitations. Firstly, this was a single-centre, retrospective, observational study. We included patients who had at least one TCD value, as some were too ill and died before a second scan could be done, while others improved rapidly and had neuroprotection stopped before a follow-up scan. We did not evaluate the association between neuromonitoring and the grade of hepatic encephalopathy (HE), as the majority of patients were intubated and had Grade 4 HE at presentation, limiting clinical differentiation. As the majority of patients were either hemodynamically unstable for transfer or showed no clinical signs of raised intracranial pressure, standard practice was that CT scans were not performed. Most of the neuromonitoring was performed in line with the institution’s neuromonitoring protocol; however, some sick patients might have more undergone more frequent readings than others, thus introducing selection bias. Additionally, we did not assess the influence of neuroprotective interventions on neuromonitoring, and we did not examine the role of other neuromonitoring techniques (such as near-infrared spectroscopy) or correlation with invasive neuromonitoring techniques. We did not compare characteristics for patients who received TCD or SjVO_2_ monitoring against those who were not monitored, so differences in patient selection (selection bias) cannot be excluded. Neuromonitoring data were only collected during the first seven days, and therefore we could not determine whether the most severe abnormalities occurred closer to death or later in the disease course. Patients who continued to deteriorate beyond this window may have developed more significant changes that were not captured in our study. Existing studies in other cohorts with brain injury suggest that TCD is useful not only for predicting death but can also confirm brain death with high accuracy [27–29]. Lastly, we did not report long-term follow-up of these children from a neurologic perspective.

This study may be relevant to paediatric patients with intracranial hypertension of varying aetiologies. However, further research with larger sample sizes is necessary to generalize these results. The findings in our study relate specifically to mortality and do not necessarily reflect long-term neurological outcomes. Therefore, future studies should investigate the impact of neuroprotective measures on neuromonitoring parameters and assess how these measures influence subsequent neurological recovery.

## Conclusion

In this retrospective cohort study of critically ill children with PALF, we describe the association of bedside neuro-monitoring modalities with clinical outcomes, including survival. Our findings demonstrated that patients who died had a high RMCA PI and lower Vm, as well as lower SjvO_2_. Paediatric intensivists could consider the use of TCD and SjvO_2_ as useful adjuncts for the comprehensive neurological assessment in critically ill children with PALF.

## Supplementary Information

Below is the link to the electronic supplementary material.

ESM 1 Supplementary Material 1 (DOCX 107 KB)

## Data Availability

No datasets were generated or analysed during the current study.
