# Supplementary material for: Utility of transcranial Doppler and reversed jugular venous saturations for neuromonitoring in children with acute liver failure
Source: Eur J Pediatr. 2025 Dec 24;185(1):39. doi: 10.1007/s00431-025-06697-2 (PMC12738604; doi:10.1007/s00431-025-06697-2)
Supplement: Supplementary file 1 — Supplementary Material 1 (DOCX 107 KB) [file 431_2025_6697_MOESM1_ESM.docx]

**SUPPLEMENTARY MATERIALS**

**Utility of Transcranial Doppler and Reversed Jugular Venous Saturations for Neuromonitoring in Children with Acute Liver Failure**

**Supplementary** **Figure 1: Correlation between RMCA PI and SjvO2**

SjvO2: Reverse jugular venous saturation; RMCA PI: Right middle cerebral artery pulsatility index

For each data point, the values are taken within a time frame of 30 minutes

Correlation r: -0.31

p-value: 0.006

**Supplementary Figure 2: Correlation between RMCA Vm and SjvO2**

Correlation r: 0.40

p-value: 0.0004

SjvO2: Reverse jugular venous saturation; RMCA Vm: Right middle cerebral artery mean velocity

For each data point, the values are taken within a time frame of 30 minutes

**Supplementary Table 1: Neuroprotection Protocol**

| - Elevate head of bed at 15˚-30˚ - Maintain neck in neutral position - Sedation :   - Adequate Sedation (opiods and benzodiazepines) ± Paralysis   - Fentanyl / Propofol boluses pre-procedure/pre-suction/movement/physiotherapy - Body Temperature : Normothermia (36.5˚ C– 37.5˚ C) – Aggressively treat hypothermia or hyperthermia - Aim for PaCO2 between 4.5-5.2 kpa - Hyperventilation : To be avoided except when there is impending herniation and used only temporarily - Blood Pressure : Maintain BP > 50th centile for age (use vasopressors if required) - Fluids :   - 2/3rd maintainence   - Maintain normoglycaemia ( 4-8 millimoles/L)   - Maintain serum sodium between 145-150 meq/l.   - 2.7% hypertonic saline infusion (0.1-1ml/kg/hr) - Consider antiepileptics if clinical (obvious seizures, unexplained tachycardia, hypertension, pupillary changes) or EEG evidence of seizures - Mannitol :   - Dose : 0.5gm/kg bolus ONLY   - Indication : Only if impending herniation and serum osmolality < 320 mosm/litre |
| --- |
